# Validity and Reliability of the Baby and Child Eating Behavior Questionnaire, Toddler Version (BEBQ-Mex and CEBQ-T-Mex) in a Low Sociodemographic Sample Recruited in a Mexican Hospital

**DOI:** 10.3390/bs11120168

**Published:** 2021-12-02

**Authors:** Claudia Hunot-Alexander, Jocelyn González-Toribio, Edgar Manuel Vásquez-Garibay, Alfredo Larrosa-Haro, Erika Casillas-Toral, Carmen Patricia Curiel-Curiel

**Affiliations:** 1 Instituto de Nutrición Humana, Centro Universitario de Ciencias de la Salud, Universidad de Guadalajara, Sierra Mojada 950, Col. Independencia, Guadalajara C.P. 44340, Jalisco, Mexico; claudia.hunot@academicos.udg.mx (C.H.-A.); jocelyn.gonzalez7445@alumnos.udg.mx (J.G.-T.); alfredo.larrosa@academicos.udg.mx (A.L.-H.); patriciacurielc@gmail.com (C.P.C.-C.); 2 Hospital Civil “Dr. Juan I. Menchaca”, Salvador Quevedo y Zubieta 750, Guadalajara CP 44340, Jalisco, Mexico; erika.casillas@academicos.udg.mx

**Keywords:** infants, toddlers, appetitive traits, appetite, validity, reliability

## Abstract

The objective of this study was to validate and measure the internal reliability of the Baby and Child Eating Behavior Questionnaires for Toddlers (BEBQ-Mex and CEBQ-T-Mex), that evaluate appetitive trait (ATs). Mothers recruited from a public hospital in Guadalajara, Mexico, completed the BEBQ-Mex or CEBQ-T-Mex along with information on sociodemographic characteristics. Internal reliability of the BEBQ-Mex was sufficient for Food Responsiveness (FR) (Cronbach α = 0.82), while Enjoyment of Food (EF) and Satiety Responsiveness (SR) showed poor reliability (α = 0.56) and Slowness in Eating (SE) had unacceptable reliability (a = 0.36). All reliability values for the CEBQ-T-Mex were acceptable (>0.70), except for SE (α = 0.64). Confirmatory factor analysis (CFA) revealed an adequate model fit for the BEBQ-Mex, except the SE subscale. CFA for the CEBQ-T-Mex confirmed the six-factor structure. Mothers of a low sociodemographic background were unable to recognize their infants’ ATs; the BEBQ-Mex partly met the criteria for validity and reliability. Mothers from similar sociodemographic characteristics were more able to recognize the ATs of their toddlers than their infants; the CEBQ-T-Mex was found to be a valid and reliable tool. Findings support the need to help mothers’ ability to recognize their infants’ ATs, which have been previously associated with weight and growth.

## 1. Introduction

Each individual is born with certain genetic characteristics, and these can be susceptible to the environment in which they interact, including the first 1000 days of life [1]. Both the infant during its first six months of life and the toddler from the ages of one to three years old, represent stages of the life cycle that could lead to the development of overweight or obesity or stunted growth and could be used as stages to design prevention interventions [2]. The prevalence of obesity has increased considerably worldwide over the last 20 years and is a major public health concern [3]; in 2016, 41 million children under the age of five were found to be overweight or obese [4]. In Mexico, results from the 2018 National Health and Nutrition Survey showed a risk of children under four years of age of becoming overweight of 22.2% and a prevalence of 8.2% [5]. Furthermore, despite the implementation of programs and actions aimed at reducing the prevalence of malnutrition, no reduction was observed for this indicator in the past few years (13.6% in 2012 vs. 14.2% in 2018–2019) [5].

Certain eating behavior dimensions have been associated with the development of overweight and obesity, as well as failure to thrive [6]. These can be described as genetic predispositions towards foods that interact with the environment to influence eating and weight gain [7], known as appetitive traits [8,9,10]. The Behavioral Susceptibility Theory (BST), proposes that there are both genetic and environmental risks that determine the responsiveness of each individual to opportunities to eat, measured through appetitive traits [11,12]. The proclivity towards a greater or lower response to food or satiety sensitivity could lead each individual to increase their weight, or to not gain weight if they have a poorer appetite, from an early age [6,9,10].

These findings have been studied in longitudinal twin cohorts [13,14], which have allowed the impact of genetic and environmental aspects to be studied in individuals who share either half or 100% of their genes [11,13]. These appetitive traits that have been related to unhealthy weight trajectories can be measured via psychometric tools.

The first questionnaire to be developed was the Child Eating Behavior Questionnaire (CEBQ), which measures eight parent-reported appetite traits in children aged three to 13 years [9]. The CEBQ is a reliable questionnaire translated into 14 languages and targets associations between several appetite traits and adiposity, both cross-sectionally and over time [6]. The CEBQ has been adapted to measure these traits in infants from zero to six months old who are exclusively milk-fed through the Baby Eating Behavior Questionnaire (BEBQ), which was examined for validation in a sample of 2402 families of twins [14]. In order to measure these traits during toddlerhood (1 to 3 years), it was adapted as the Children’s Eating Behavior Questionnaire for toddlers (CEBQ-T) to describe the onset of emotional eating at this stage of life [15,16], and the importance of the introduction to family’s food environment, with its potential consequences.

The BEBQ and CEBQ-T could inform trends towards higher food consumption through food approach or avid appetite subscales (Food Responsiveness, Enjoyment of Food, Emotional Overeating) or towards a lower food consumption through food avoidance subscales (Satiety Responsiveness, Food Fussiness, Slowness in Eating). Both of these questionnaires could be used to manage behavioral strategies tailored to an infant or toddler’s appetitive trait profile, as has been carried out in adults using the Adult Eating Behavior Questionnaire [17]. Neither the BEBQ nor the CEBQ-T have been examined for validation in México. Some results of the relationship between appetitive traits and body mass index (BMI) in infants under six months of age have been studied in Mexico, but no validation of the questionnaires has been published [18]. Thus, the primary aims of this research were (1) to translate and confirm the structure of the BEBQ and CEBQ-T into Spanish in a Mexican population of infants and toddlers attending a large public hospital in Guadalajara, Mexico, and (2) to determine the internal reliability of both instruments. As a secondary aim, we explored the associations between appetitive traits for the BEBQ-Mex and the CEBQ-T-Mex and the associations between the appetite traits of infants and toddlers with the BMI z-scores (BMI-z) of the sample.

## 2. Materials and Methods

### 2.1. Translation of the Questionnaires and Use of the “Think Aloud” Methodology

This study was cross-sectional and analytical. Both questionnaires (the BEBQ and the CEBQ-T) were translated into Spanish by a bilingual researcher (C.H.A.) and reviewed by other authors (E.V.G. and P.C.C.) and other translators. Recommendations by the World Health Organization were followed, using forwards and backwards translation [19].

To ensure that the questionnaires were understood and provide face validity, a sample of 10 mothers of infants and 10 mothers of toddlers were interviewed. Cognitive structured interviews were conducted using the “Think-Aloud” methodology [20,21]. This methodology allows researchers to establish the level of understanding that the participants have of the items of the questionnaire and is said to provide a valid reflection of individuals’ thoughts in response to a question or task. These interviews were conducted by two standardized professionals with experience in eating behavior data collection (J.G.T., P.C.C.). The mothers were asked to read the questions aloud and to comment on their understanding of the question, as well as their mental reasoning process underlying each response. All the interviews were recorded using a digital audio recorder and transcribed manually to analyze responses.

### 2.2. Data Collection

Once the questionnaires were deemed to be understood by the mothers, the sample size was calculated to show the significance of Pearson’s correlation coefficients between appetite traits and BMI-z, giving a result of 330 infants (0 to 12 months old) and 330 toddlers (12 to 36 months of age) [22]. Participants were recruited at the outpatient department of the Pediatric Division of the Hospital Civil de Guadalajara “Dr. Juan I Menchaca” during the period of 2018–2020. This area of the hospital facilitates access to healthy children to receive food guidance and immunizations. Those children whose mothers agreed to sign the informed consent form were included in the study through consecutive case sampling. Data were collected by J.G.T., supervised by P.C.C. as well a team of dietitians who were standardized to apply the questionnaire to the mother, explaining with examples of possible situations in each of the items of both instruments. J.G.T. supervised the completion of the questionnaires, so there were no missing data to handle. The main non-inclusion criteria were children with diseases that could modify their appetites, such as acute respiratory or digestive diseases of more than a week, malnutrition, major congenital malformations, or chronic diseases, as well as infants and toddlers who did not attend the hospital with their mothers.

### 2.3. Sociodemographic Variables

Sociodemographic data were collected and included maternal level of education (basic level, minimum of six years of schooling or less; secondary education, minimum of a baccalaureate or technical level of education; professional education, bachelor’s education or more); mother’s occupation (housewife or employed); marital status (in partnership, married or cohabiting; single, single, divorced, separated, or widowed). In addition, family type was examined and classified as nuclear when only parents and children lived under the same roof and as ‘other’ when there was someone else living at home. Likewise, monthly income was asked, classifying it as equal to or less than 4500 Mexican pesos (MXN) or more, which is equivalent to 226 US dollars (approximately 20 pesos/US dollar in September 2021). The data collected for the infants and toddlers were age, sex, feeding type in the first six months of life (exclusive breastfeeding; partial breastfeeding; human milk substitutes), and whether a carer different from the parents was in charge of the child (yes; no).

### 2.4. Anthropometry

Anthropometric data were collected in both groups. Infants were weighed and measured with a scale and Infantometer ^®^SECA 334 (GmbH & Co. KG, Hamburg, Germany). Toddlers were weighed with a ^®^Tanita scale (Tanita, Tokyo, Japan), and height over two years of age was measured using a stadiometer ^®^SECA 213 (GmbH & Co. KG, Hamburg, Germany). Length and height were measured in centimeters and weight in grams. These measurements were used to calculate BMI-z and classify them according to WHO standards (normal weight: BMI-z = 0.9–1.9; overweight: BMI-z = 2.0–2.9; obesity: BMI-z ≥ 3) [23].

### 2.5. Feeding Type

We were interested in knowing if the mothers had exclusively breastfed, had given human milk substitute or partial breastfeeding to her child. We only interviewed mothers whose infants were older than four months of age, as this could be within a closer range to the six-month threshold set by the WHO and account for exclusive breastfeeding.

### 2.6. BEBQ-Mex and CEBQ-T-Mex Questionnaires

Infants’ mothers answered the 18 items that make up the BEBQ-Mex according to the two versions of the questionnaire [14]: (1) the concurrent version for mothers whose baby was still exclusively breastfeeding, and (2) the retrospective version for mothers whose baby had started complementary feeding from 6 to 12 months of age. The BEBQ-Mex measures two food approach subscales (Food Responsiveness and Enjoyment of Food), two food avoidance subscales (Satiety Responsiveness and Slowness in Eating), and one General Appetite item.

The mothers of the toddlers answered 26 items that make up the CEBQ-T-Mex [15]. The CEBQ-T-Mex measures three food approach subscales (Food Responsiveness, Enjoyment of Food, and Emotional Overeating) and three food avoidance subscales (Satiety Responsiveness, Food Fussiness, and Slowness in Eating). Both questionnaires have a Likert response scale of 1 to 5 (1 = never, 2 = rarely, 3 = sometimes, 4 = often, 5 = always).

### 2.7. Reliability and Validation Measures of the Questionnaires

IBM SPSS version 25 for Windows was used to test the internal reliability of each of the BEBQ-Mex and CEBQ-T-Mex subscales, which were examined using Cronbach’s alpha, (>0.9, excellent; 0.80–0.89, good; 0.70–0.79, acceptable; 0.60–0.69 questionable; 0.50–0.59, poor) [24]. We also calculated Omega coefficients to remove potential errors in reliability estimation [25]. Omega coefficients greater than or equal to 0.70 were also considered reliable [25]. We also examined the internal reliability of both questionnaires by sex and feeding type.

Confirmatory factor analysis (CFA) was used to validate the questionnaires. Normative chi-square values (χ^2^/degrees of freedom) between 1.0 and 2.0 or 2.0 and 3.0 indicate a good or acceptable model fit, respectively [26,27]. Goodness-of-fit indices were also measured using the Comparative Fit Index (CFI), the Norm Fit Index (NFI), the Tucker–Lewis index (TLI), and the mean square error of approximation (RMSEA). CFI and NFI values of greater than 0.9 and RMSEA values less than 0.08 are considered good model fit indices [27]. Residual variances, factor loadings of the items, factor variances, item variances, and modification indices were also taken into account to analyze the model fit. We tested two different models for the BEBQ-Mex: (1) Model 1, which included all items, and (2) Model 2, where we made adjustments to three covariance errors detected to see if we could improve the model-based modification indices analysis. JASP 0.14 statistical program was used to carry out the CFA and Omegas [28].

### 2.8. Statistical Analysis

The means and standard deviations of each subscale were calculated according to the following guidelines (https://www.ucl.ac.uk/epidemiology-health-care/research/behavioural-science-and-health/resources/questionnaires/eating-behaviour-questionnaires#bebqcv (accessed on 29 November 2021)). Given that the distributions of neither the BEBQ-Mex nor the CEBQ-T-Mex subscales were normal, we also present the median and ranges. We conducted non-parametric test on all samples and obtained very similar results to those with parametric tests (results not shown). However, due to the sample size and based on the central theorem, parametric tests were used [29] in order to compare our results to the way the literature is currently presented [6]. As part of the secondary aims, Pearson correlations were used to show the relationship between the subscales of each questionnaire; and linear regression analysis was used to test the associations between BMI-z (as the independent variable) and each appetite trait (as the dependent variable). Adjustments were made for BMI-z for sex, age and feeding type in the first six months of life for both infants and toddlers.

## 3. Results

### 3.1. Translation and “Think Aloud” Methodology

Ten mothers of infants who were between the ages of 19 and 40 years (28.4 + 6.3) and 10 mothers of toddlers between the ages of 19 and 38 years (26.8 + 6.1) were involved in validating the translation of the questionnaires. All the participants had a minimum of primary school education. Participants found all items to be adequate and showed understanding of both questionnaires. The final BEBQ-Mex and CEBQ-T-Mex versions are shown in Supplementary Materials Information S1.

### 3.2. Sociodemographic Characteristics

Sociodemographic characteristics were collected from 330 mother–infant dyads and 330 mother–toddler dyads. The mothers and families of age groups shared almost identical sociodemographic characteristics. A basic level of education prevailed (52.8%), the main occupation of the mothers was being a housewife or unemployed (75.8%), children were often raised in a nuclear family (55%), and the monthly family income was below MXN 4500, equivalent to less than USD 226. Regarding infants and toddlers, 49.7% and 55.8% were male and 50.3% and 44.2% were female, respectively. Despite the homogeneous characteristics in the data, this sample only represents a particular group of the Mexican population due to the different socio-cultural and socioeconomic strata that exist in this country. Approximately 40% and 45% of infants and toddlers were exclusively breastfed in the first four to six months of life. Most were found in a normal BMI-z range (75.5% and 70.9%, respectively) (Table 1).

### 3.3. Descriptive Statistics and Internal Reliability of the BEBQ-Mex and CEBQ-T-Mex

Mean and standard deviation scores and internal reliability estimates are presented in Table 2 for both the BEBQ-Mex and the CEBQ-T-Mex. The internal reliability results for the BEBQ-Mex for all subscales were all below 0.70 (Cronbach’s alpha and omega), except for Food Responsiveness (α = 0.82, ω = 0.82). Both Enjoyment of Food and Satiety Responsiveness showed poor Cronbach alpha and omega values (α = 0.56, ω = 0.56, respectively), and Slowness in Eating showed an unacceptable internal reliability (α = 0.36, ω = 0.49). Internal reliability values for both Cronbach alpha and omega values were acceptable (>0.70) for all CEBQ-T-Mex subscales, except for Slowness in Eating, which was lower (α = 0.64, ω = 0.66) (Table 2).

Table 3 shows the internal reliability of the subscales for both the BEBQ-Mex and the CEBQ-T-Mex subscales by sex and feeding type in the first six months of life. The BEBQ-Mex showed similar Cronbach alphas by sex for all subscales. Reliabilities by feeding type were lower in those who received partial breastfeeding for Enjoyment of Food, Satiety Responsiveness, and Slowness in Eating in the BEBQ-Mex group (α = 0.36, α = 0.38, α = 0.32, respectively), and were lower in those receiving human milk substitutes in for Emotional Overeating, Satiety Responsiveness, Food Fussiness, and Slowness in Eating for the CEBQ-T-Mex (α = 0.55, α = 0.63, α = 0.81, α = 0.60, respectively).

### 3.4. Confirmatory Factor Analysis

Table 4 shows the results of the CFA. In the first BEBQ-Mex model, all four original factors (17 items, excluding one General Appetite item) were included in the results, where Χ^2^/degrees of freedom = 1.92; RMSEA = 0.05; CFI = 0.90; NFI = 0.82; TLI = 0.88, all showing good to acceptable model fits. All the variances and factor loadings were significant (*p* < 0.05) with the exception of item 15 (*p* = 0.14) (Figure S1). Most of the results of the standardized regressions of the items were greater than 0.3 except for items 3 (0.25), 6 (0.24), and 15 (0.13). The R^2^ value of the items were above 0.1 except for items 5 (0.08), 6 (0.06), and 15 (0.00). Thus, despite results from Model 1, we decided not to eliminate any of the items and test the model again in a second model using modification indices for the three covariance adjustments detected to see if we could improve the model (30). The covariance errors were added between items 12 and 18 of the Food Responsiveness factor (subscale), 5 and 15 in the Slowness in Eating factor (subscales), and 6 and 17 in the Enjoyment of Eating factor (subscale), due to the similarity in the theoretical content of the coupled items, correlating that the error of the terms is considered justifiable (30). Results for Model 2 shown in Table 4 again all showed good to acceptable model fits: Χ^2^ = 1.65; RMSEA = 0.04; CFI = 0.95; NFI = 0.85; TLI = 0.91. All the variances and factor loadings remained significant (*p* < 0.05), and all the model structures are presented in Supplementary Materials Information S2, Figure S1. The results for the CEBQ-T-Mex showed a good to acceptable model fit: Χ^2^/degrees of freedom = 2.78; RMSEA = 0.074; CFI = 0.84; NFI = 0.80. All the variances and factor loadings were significant (*p* < 0.05) (Supplementary Materials Information S2, Figure S2).

### 3.5. Associations between BEBQ-Mex and CEBQ-T-Mex Subscales

In the BEBQ-Mex, no significant correlations were found between the food approach subscales. Enjoyment of Food was inversely correlated to all food avoidance traits and all food avoidance traits were positively and significantly correlated between each other (Table 5). For the CEBQ-T-Mex, food approach subscales were positively and significantly correlated to each. Food approach subscales were negatively correlated to the food avoidance subscales of Satiety Responsiveness, and only Food Responsiveness and Enjoyment of Food were negatively correlated to Food Fussiness. Food avoidance subscales were positively correlated with each other except with Food Fussiness and Slowness Eating (Table 6).

### 3.6. Associations between BEBQ-Mex and CEBQ-T-Mex and BMI-z Scores

Results from the linear regressions between appetitive traits and BMI-z, which can be found in the Supplementary Materials Information S2, Table S4, showed that only the General Appetite item of the BEBQ-Mex was positively associated with the BMI-z of the infant BMI [β = 0.10 * (0.02, 0.18)]. After adjusting for sex, age, and feeding type, BMI-z was negatively associated with Slowness in Eating [β = −0.07 * (−0.13, −0.001)] and positively associated with General Appetite [β = 0.09 * (0.01, 0.17)], as expected (Table S1). On the other hand, for the CEBQ-T-Mex after adjusting for sex, age, and feeding type, BMI-z was directly associated with Enjoyment of Food [β = 0.13 ** (0.03, 0.22)] and negatively associated with Satiety Responsiveness [β = −0.15 ** (−0.24. −0.05)] and Food Fussiness [β = −0.07 ** (−0.17, −0.04)], as expected (Table S1).

## 4. Discussion

This study aimed to adapt the factor structure of the BEBQ and the CEBQ-T in a Mexican population recruited in a pediatric outpatient department of a public hospital in Guadalajara, México. The validity of the BEBQ-Mex showed discrepancies using the four subscales proposed in the original version of the BEBQ [14]. Adjustments were made by adding three covariance errors in three different factors in a second model. Similar covaried errors for Satiety Responsiveness were used in a validation study of the BEBQ in a sample of 467 Australian infants aged 17 weeks ± 3 weeks, [26]; our differences were found with Slowness in Eating. These results also appeared to also affect the internal reliability analysis for this construct.

CFA for the CEBQ-T-Mex using the original six factor version of the CEBQ-T [10,15] showed an adequate validity (RMSEA = 0.07) [30]. Based on the study of 2402 twin pairs born in England and Wales in 2007, who first completed the BEBQ and years later the CEBQ, Herle analyzed through structural equation modelling the heritability of Emotional Overeating and found it to be very low, specifically influenced by the environment in which the child develops, mainly by parents [16]. At this age, the child also starts to develop Food Fussiness as an eating behavior dimension that has been found to be highly heritable and an important contributor to poorer diet quality in the toddler [31].

The reliability of the BEBQ-Mex supports the usefulness of the Food Responsiveness subscale (α = 0.82, ω = 0.82). Enjoyment of Food (α = 0.56, ω = 0.56) and Satiety Responsiveness (α = 0.56, ω = 0.56) were considered poor, and Slowness in Eating (α = 0.36, ω = 0.49) was unacceptable, unlike the original BEBQ, where all four subscales obtained internal reliability values above 0.70 [14]. In the Australian BEBQ validity study, the Satiety Response subscale was deemed adequate (α = 0.56) [26]. The reliability of the CEBQ-T-Mex showed good and acceptable results (α > 0.7, ω = 0.7), except for the Slowness in Eating (α = 0.64, ω = 0.64).

When analyzing internal reliability by sex and feeding type for each subscale for both questionnaires, Emotional Overeating tended to be lower in toddler males than females (α = 0.60 vs. α = 0.78). These differences were not so apparent by sex in infants for any of the subscales. In general, there is a greater reliability of the BEBQ, when the child is exclusively breastfed. Appetite-regulating hormones in infants vary depending on the type of feeding they receive [32], and when they are exclusively breastfed, they regulate intake during the feed [33]. For the CEBQ-T-Mex, Emotional Overeating had a lower reliability in toddlers who have been fed human milk substitutes compared to exclusive breastfeeding and partial breastfeeding during the first six months of life (α = 0.55, α = 0.78, and α = 0.72, respectively). Satiety Responsiveness also showed a lower reliability in toddlers who were fed human milk substitutes compared to partial breastfed or exclusively breastfed (α = 0.63, α = 0.77, and α = 0.78, respectively). These results differ in the Australian validity study of the BEBQ, where Satiety Responsiveness was lower in infants who were exclusively breastfeed compared to non-breastfed infants (α = 0.50 vs. α = 0.68) [26], unlike the results of the original BEBQ study where the majority of results by feeding type were found to have good internal reliability (α > 0.7) [14]. There is evidence that mothers are more responsive to their child’s hunger than to their fullness cues, as it appears we are evolutionarily set up to protect growth and stave off hunger [34]. This could explain the differences observed in Satiety Responsiveness reliability by feeding types. Moreover, higher education has been shown to be associated with responsiveness to feeding cues, perhaps due to a potential recognition of the importance of child development [34], contrary to the low socioeconomic level of the mothers in our study.

Although the BEBQ-Mex was not found to be reliable and validity was partly shown compared to the CEBQ-Mex, we consider this to be an important finding. Appetite from the beginning of life has been shown to be associated with weight and infant growth [6]. This has been shown in twin studies, where twins with more avid appetites have been found to have higher weights than those who do not. Other studies have shown that a low appetite is also associated with a decrease in growth and leads to smaller babies [13]. The fact that the CEBQ-T-Mex is a valid and reliable measure and that the data obtained for both questionnaires come from mothers with very similar sociodemographic characteristics suggests that these traits are not recognized by the mothers in their infants, which could lead to problems with the weights of infants and into toddlerhood. In this population, the capacity to read their infants’ appetites appears to start once the infants have begun their complementary feeding. Efforts should be made to start a responsive feeding program in this population [2]. Mexico is characterized by a large percentage of its population (60%) in this sociodemographic group [35]. Given the increase in overweight and obesity in these age groups, understanding how infants’ different types of appetite affect their weight is essential for prevention programs. Timely interventions in the first 1000 days of life are key to metabolic programming of the individual [36].

Correlations between the subscales for each questionnaire showed varying results. Significant correlations between the food approach subscales were expected as seen in other studies [13,37,38], but not seen in the BEBQ-Mex. Food avoidance subscales were positively correlated to each other, as expected. Correlations between food approach and food avoidance subscales were mainly inverse (*p* < 0.001), as expected. The General Appetite item was directly correlated with the food approach subscales and with the Satiety Responsiveness subscales, suggesting that the greater the appetite, the greater the level of satiety, but inversely correlated with the Slowness in Eating subscale, as expected (*p* < 0.001). In the CEBQ-T-Mex, the food approach subscales were positively correlated with each other (*p* < 0.001), and the food avoidance subscales were also positively correlated between each other (*p* < 0.001), except for Food Fussiness with Slowness in Eating. Again, these results highlight the difficulties in understanding the Slowness in Eating dimension. Mothers do not appear to understand the concept of a perceptive or responsive type feeding [2,36,39], where they are able to distinguish when their child continues to eat more food at a faster rate despite being full, without noticing when they are satisfied [31], potentially losing their ability to self-regulate her own appetite [2].

Although the questionnaires do not meet the requirements to be validated, we consider it important to discuss the results of the correlations between the BEBQ-Mex, CEBQ-T-Mex, and BMI-z subscales because they are in indirect opposition to the results found in different studies around the world [13,34,37], possibly pointing to a misinterpretation of the child’s external hunger and satiety cues.

This is a novel study of the measurement of appetite traits via the use of simple and inexpensive questionnaires from 330 infants and 330 toddlers associated with higher adiposity. However, it has several limitations. The participants were obtained from a public hospital and their mothers had homogenous sociodemographic characteristics, education levels, and average family monthly incomes [40]. Further data should be collected in diverse populations to see if there is a better understanding of the children’s appetitive traits, more so in infants regarding Enjoyment of Food, Satiety Responsiveness, and Slowness in Eating. Qualitative studies would also allow in-depth research into the discourses of mothers’ eating behavior dimensions of their infants and toddlers. Generalization of the results can only be limited to the cultural and socioeconomic level of the mothers who answered the questionnaires, and external reliability studies should be included. However, these results point towards the importance of helping mothers, particularly of infants, to recognize their children’s hunger and satiety cues, which have been previously associated with changes in nutritional status [2,37].

## 5. Conclusions

In conclusion, this study examined the validity and internal reliability of the BEBQ-Mex and CEBQ-T-Mex measures of appetitive traits, in a population of lower socio-demographic-background infants and toddlers who attended a pediatric outpatient clinic at a Mexican public hospital. Even though the BEBQ-Mex did not meet the established criteria for its validity and reliability, we consider that its structure must be maintained for comparisons in future studies. Once measurements were made in toddlers, the mothers appear to be more able to recognize these appetitive traits in their children and thus, the CEBQ-T-Mex was found to be a valid and reliable tool. Both these questionnaires serve as measures to generate awareness in Mexican mothers towards different eating behavior dimensions that are possibly associated with the development of obesity and to potentially generate timely interventions in the first 1000 days of life.

## Figures and Tables

**Table 1:** Sociodemographic characteristics of infants (n = 330) and toddlers (n = 330) and their respective families.

Characteristics	Infants	Toddler
**Maternal**	n = 330 MD (SD)	n = 330 MD (SD)
Age (years)	28.45 (6.27) n (%)	26.77 (6.12) n (%)
BMI (kg/m^2^)		
Healthy weight	152 (46.2)	148 (44.8)
Overweight	100 (30.4)	101 (30.6)
Obese	77 (23.4)	81 (24.6)
Education		
Basic education	196 (52.8)	199 (60.3)
Secondary education	109 (33.2)	92 (27.9)
Professional education	46 (14.0)	39 (11.8)
Occupation		
Housewife/Unemployed	250 (75.8)	224 (68.0)
Employed	80 (24.2)	105 (32.0)
Marital status		
In partnership	276 (84.4)	266 (80.6)
Single	51 (15.6)	64 (19.4)
Family type		
Nuclear	181 (55.0)	208 (63.0)
Other ^1^	148 (45.0)	122 (37.0)
Monthly family income ^2^		
<MXN 4500	262 (82.4)	265 (82.6)
>MXN 4500	56 (17.6)	56 (17.4)
**Children**	MD(SD)	MD (SD)
Age (months)	7.60 (2.10) n (%)	20.98 (6.53) n (%)
Sex		
Male	164(49.7)	184 (55.8)
Female	166(50.3)	146 (44.2)
Weight (kg)	7.66 (1.27)	10.94 (1.95)
Height (cm)	67.30 (4.51)	81.14 (6.37)
z-BMI ^3^		
Low weight	28 (8.5)	8 (2.5)
Normal weight	285 (86.5)	299 (91.4)
Overweight	14 (4.40)	9 (2.8)
Obese	2 (0.60)	11 (3.3)
Feeding type		
Exclusive breastfeeding	133 (40.0)	148 (45.0)
Mixed feeding	108 (33.0)	113 (34.0)
Human milk substitute	88 (27.0)	69 (21.0)
Carer different from parents ^4^		
No	246 (76.2)	224 (68.0)
Yes	77 (23.8)	105 (32.0)

^1^ Monoparental, extended, joint. ^2^ Mexican pesos. ^3^ WHO. Normal weight: normal weight: z-BMI = 0.9–1.9; overweight: z-BMI = 1.9–2.9; obesity: z-BMI ≥ 3. ^4^ Mothers were asked if someone other than the parents looked after the child.

**Table 2:** Internal reliability (Cronbach’s alpha and omega) of the BEBQ-Mex (n = 330) and the CEBQ-T- Mex (n = 330).

Appetitive Traits		BEBQ-Mex					CEBQ-T-Mex			
	Mean ± SD	Median (IR)	Cronbach α (ω)	Omega ω		Mean ± SD	Median (IR)	Cronbach α (ω)	Omega ω	
				IC	SC				IC	SC
	Food approach subscales									
Food Responsiveness	2.80 ± 1.14	2.60 (1.83)	0.82 (0.82)	0.79	0.85	2.54 ± 1.21	2.20 (2.00)	0.80 (0.80)	0.76	0.83
Emotional Overeating		NA				1.40 ± 0.65	1.00 (0.67)	0.71 (0.71)	0.65	0.76
Enjoyment of Food	4.70 ± 0.46	5.00 (0.50)	0.56 (0.56)	0.47	0.63	3.88 ± 0.95	4.00 (1.50)	0.79 (0.79)	0.76	0.83
	Food avoidance subscales									
Satiety Responsiveness	2.12 ± 0.92	2.00 (1.33)	0.56 (0.56)	0.47	0.64	2.76 ± 0.97	2.80 (1.40)	0.74 (0.74)	0.70	0.79
Food Fussiness		NA				2.40 ± 1.07	2.10 (1.67)	0.84 (0.84)	0.81	0.86
Slowness in Eating	2.17 ± 0.79	2.00 (1.25)	0.36 (0.49)	0.37	0.57	2.99 ± 0.54	3.00 (0.50)	0.64 (0.66)	0.60	0.72

IR: interquartile range; IC: inferior confidence intervals 95.0%; SC: superior confidence intervals 95%; NA: not applicable; BEBQ-Mex: Baby Eating Behavior Questionnaire, Mexican version; CEBQ-T-Mex: Child Eating Behavior Questionnaire-Toddler, Mexican version.

**Table 3:** Internal reliability (Cronbach’s alpha) by sex and feeding type of the BEBQ-Mex (n = 330) and the CEBQ-T-Mex (n = 330).

Sample	Cronbach’s Alpha (n)									
	Food Responsiveness		Emotional Overeating	Enjoyment of Food		Satiety Responsiveness		Food Fussiness	Slowness in Eating	
	BEBQ	CEBQ-T	CEBQ-T	BEBQ	CEBQ-T	BEBQ	CEBQ-T	CEBQ-T	BEBQ	CEBQ-T
Total (n)	0.82 (329)	0.80 (329)	0.71 (328)	0.55 (328)	0.79 (329)	0.56 (328)	0.74 (330)	0.84 (328)	0.36 (328)	0.64 (330)
Male (n)	0.81 (163)	0.78 (183)	0.60 (183)	0.59 (162)	0.80 (184)	0.54 (162)	0.76 (184)	0.85 (182)	0.35 (162)	0.67 (184)
Female (n)	0.83 (166)	0.81 (146)	0.78 (145)	0.52 (166)	0.78 (145)	0.56 (166)	0.71 (146)	0.82 (146)	0.39 (166)	0.61 (146)
EBF (n)	0.81 (133)	0.77 (147)	0.78 (148)	0.52 (132)	0.75 (148)	0.59 (132)	0.76 (148)	0.85 (148)	0.34 (133)	0.64 (148)
PBF (n)	0.82 (108)	0.83 (113)	0.72 (112)	0.36 (108)	0.84 (112)	0.38 (107)	0.77 (113)	0.85 (111)	0.32 (108)	0.70 (113)
HMS (n)	0.83 (87)	0.78 (69)	0.55 (68)	0.70 (87)	0.77 (69)	0.59 (88)	0.63 (69)	0.81 (69)	0.37 (86)	0.60 (69)

EBF: exclusive breastfeeding; PBF: partial breastfeeding; HMS: human milk substitute; BEBQ-Mex: Baby Eating Behavior Questionnaire, Mexican version; CEBQ-T-Mex: Child Eating Behavior Questionnaire-Toddler, Mexican version.

**Table 4:** Confirmatory factor analysis and model fit indices of the BEBQ-Mex (n = 330) and the CEBQ-T-Mex (n = 330).

Model Fit Indices	BEBQ-Mex		CEBQ-T-Mex
	Model 1	Model 2	Model 1
	n = 330		n = 330
X^2^/(degrees of freedom)	1.92	1.65	2.78
X^2^ *p*	<0.001	<0.001	<0.001
RMSEA	0.05	0.05	0.07
CFI	0.90	0.95	0.84
NFI	0.82	0.85	0.80
TLI	0.88	0.92	0.82

CFI: comparative fit index; NFI: normed fit index; RMSEA: root mean square error of approximation; TLI: Tucker–Lewis index; BEBQ-Mex: Baby Eating Behavior Questionnaire, Mexican version; CEBQ-T-Mex: Child Eating Behavior Questionnaire-Toddler, Mexican version.

**Table 5:** Pearson’s correlations between appetitive trait subscales for the BEBQ-Mex (n = 330).

BEBQ-Mex Subscales	Food Responsiveness	Enjoyment of Food	Satiety Responsiveness	Slowness in Eating	General Appetite
Food approach subscales					
Food Responsiveness	1	−0.03	−0.10	0.02	0.29 **
Enjoyment of Food		1	−0.33 **	−0.25 **	0.33 **
Food avoidance subscales					
Satiety Responsiveness			1	0.22 **	0.37 **
Slowness in Eating				1	−0.15 **
General Appetite					1

FR: Food Responsiveness; EF: Enjoyment of Food; SR: Satiety Responsiveness; SE: Slowness in Eating; BEBQ-Mex: Baby Eating Behavior Questionnaire, Mexican version. * Correlation is significant at the 0.01 level (2-tailed). ** Correlation is significant at the 0.05 level (2-tailed).

**Table 6:** Pearson’s correlations between appetitive trait subscales for the CEBQ-T-Mex (n = 330).

CEBQ-T-Mex Subscales	FR	EOE	EF	SR	FF	SE
Food approach subscales						
Food Responsiveness	1	0.32 **	0.51 **	−0.40 **	−0.25 **	−0.03
Emotional Overeating		1	0.20 **	−0.14 *	−0.04	0.06
Enjoyment of Food			1	−0.60 **	−0.52 **	−0.07
Food avoidance subscales						
Satiety Responsiveness				1	0.42 **	0.15 **
Food Fussiness					1	0.077
Slowness in Eating						1

FR: Food Responsiveness; EOE: Emotional Overeating; EF: Enjoyment of Food; SR: Satiety Responsiveness; FF: Food Fussiness; SE: Slowness in Eating; CEBQ-T-Mex: Child Eating Behavior Questionnaire-Toddler, Mexican version. * Correlation is significant at the 0.05 level (2-tailed). ** Correlation is significant at the 0.01 level (2-tailed).

## Data Availability

The datasets used and/or analysed during the current study are available from the corresponding author upon reasonable request.

## Supplementary Materials

The following are available online at https://www.mdpi.com/article/10.3390/bs11120168/s1, File S1: Survey on eating habits and lifestyle around the Coronavirus (COVID-19) pandemic in Mexico: ESCAN-COVID19Mx surve. Table S1. Full set of BEBQ concurrent version and BEBQ-Mex items; Table S2. Full set of BEBQ retrospective version and BEBQ-Mex items; Table S3. Full set of CEBQ-T and CEBQ-T-Mex items. File S2: Figure S1: Path diagram of the four-factor models of the BEBQ-Mex with standardized estimates (factor loadings, item squares multiple correlations, and error covariances) fitted in a sample of 330 mother–infant dyads (Model 1: left panel, Model 2: right panel); Figure S2: Path diagram of the six-factor model of the CEBQ-T-Mex with standardized estimates (factor loadings, item squares multiple correlations, and error covariances) fitted in a sample of 330 mother–toddler dyads; Table S4: Linear regressions between infant and toddler appetitive traits and BMI-z adjusting for sex, age, and feeding type.
