# Supplementary material for: Validity and Reliability of the Baby and Child Eating Behavior Questionnaire, Toddler Version (BEBQ-Mex and CEBQ-T-Mex) in a Low Sociodemographic Sample Recruited in a Mexican Hospital
Source: Behav Sci (Basel). 2021 Dec 2;11(12):168. doi: 10.3390/bs11120168 (PMC8698384; doi:10.3390/bs11120168)
Supplement: Supplementary file 1 [file behavsci-11-00168-s001.zip › behavsci-1422232-supplementary.pdf]

Validity and Reliability of the Baby and Child Eating Behavior Questionnaire, Toddler Version (BEBQ-Mex and CEBQ-T-Mex) in a Low Sociodemographic Sample Recruited in a Mexican Hospital

Claudia Hunot-Alexander [1,†], Jocelyn González-Toribio [1,†], Edgar Manuel Vásquez-Garibay [1,*], Alfredo Larrosa-Haro [1], Erika Casillas-Toral [2] and Carmen Patricia Curiel-Curiel [1]

Supplementary Information S1

Table S1. Full set of BEBQ concurrent version and BEBQ-Mex items.

BEBQ [1]	Subescales		BEBQ-Mex [2]
**Food Aproach subscales; Sub-escalas pro-ingestión**			
My baby frequently wants more milk than I provide	Food Responsiveness	Respuesta a los Alimentos	Mi bebé frecuentemente quiere más leche de la que puedo darle
If allowed to, my baby would take too much milk			Si se le permitiera, mi bebe toma demasiada leche
Even when my baby has just eaten well he/she is happy to feed again if offered			Aun cuando mi bebe ha comido bien, estaría contento(a) de volver a comer si se le ofreciera
My baby is always demanding a feed			Mi bebe siempre está demandando una comida/tetada
If given the chance, my baby would always be feeding			Si le diera chance, mi bebe siempre estaría comiendo
My baby can easily take a feed within 30 minutes of the last one			Mi bebe puede fácilmente volver a comer a los 30 minutos de la última tetada/toma
My baby seems contented while feeding	Enjoyment of Food	Disfrute de los Alimentos	Mi bebé parece contento mientras come
My baby loves milk			A mi bebe le encanta la leche
My baby becomes distressed while feeding			Mi bebe se angustia/aflige mientras come*
My baby enjoys feeding time			Mi bebe disfruta la hora de comer
**Food Avoidance subescales; Sub-escalas anti-ingestión**			
My baby gets full up easily	Satiety Responsiveness	Respuesta a la Saciedad	Mi bebe se siente lleno(a) fácilmente
My baby gets full before taking all the milk I think he/she should have			Mi bebe se llena antes de tomar toda la leche que yo pensaba que debía recibir
My baby finds it difficult to manage a complete feed			A mi bebe le cuesta trabajo terminarse una tetada/una comida completa
My baby finishes feeding quickly	Slowness in Eating	Lentitud para comer	Mi bebe se termina sus tetadas/tomas rápidamente*
My baby takes more than 30 minutes to finish feeding			A mi bebe le toma más de 30 minutos terminar de comer/ su tetada
My baby feeds slowly			Mi bebe es lento para comer
My baby sucks more and more slowly during the course of a feed			Mi bebe succiona cada vez más lento durante el transcurso de una comida/tetad
My baby has a big appetite	General Appetite	Apetito	Mi bebe tiene un gran apetito

[1] Response option: Never, Rarely, Sometimes, Often, Always. [2] Opción de respuestas: Nunca, Rara vez, Algunas veces, A menudo, Siempre.

Table S2. Full set of BEBQ retrospective version and BEBQ-Mex items.

BEBQ [1]	Subescales		BEBQ-Mex [2]
**Food Aproach subscales; Sub-escalas pro-ingestión**			
My baby frequently wanted more milk than I provided<br>If allowed to, my baby would take too much milk<br>Even when my baby had just eaten well he/she was happy to feed again if offered<br>My baby was always demanding a feed<br>If given the chance, my baby would always be feeding<br>My baby could easily take a feed within 30 minutes of the last one	Food Responsiveness	Respuesta a los Alimentos	Mi bebé frecuentemente quería más leche de la que puedo darle<br>Si se le permitía, mi bebe tomaba demasiada leche<br>Aun cuando mi bebe había comido bien, estaba contento(a) de volver a comer si se le ofrecía<br>Mi bebe siempre estaba demandando una comida/tetada<br>Si le daba chance, mi bebe siempre estaba comiendo<br>Mi bebe podía fácilmente volver a comer a los 30 minutos de la última tetada/toma
My baby seemed contented while feeding<br>My baby loved milk<br>My baby became distressed while feeding<br>My baby enjoyed feeding time	Enjoyment of Food	Disfrute de los Alimentos	Mi bebé parecía contento mientras come<br>A mi bebe le encantaba la leche<br>Mi bebe se angustiaba/afligía mientras come*<br>Mi bebe disfrutaba la hora de comer
**Food Avoidance subescales; Sub-escalas anti-ingestión**			
My baby got full up easily<br>My baby got full before taking all the milk I think he/she should have<br>My baby found it difficult to manage a complete feed	Satiety Responsiveness	Respuesta a la Saciedad	Mi bebe se sentía lleno(a) fácilmente<br>Mi bebe se llenaba antes de tomar toda la leche que yo pensaba que debía recibir<br>A mi bebe le costaba trabajo terminarse una tetada/una comida completa
My baby finished feeding quickly<br>My baby took more than 30 minutes to finish feeding<br>My baby fed slowly<br>My baby sucked more and more slowly during the course of a feed	Slowness in Eating	Lentitud para comer	Mi bebe se terminaba sus tetadas/tomas rápidamente*<br>A mi bebe le tomaba más de 30 minutos terminar de comer/ su tetada<br>Mi bebe era lento para comer<br>Mi bebe succionaba cada vez más lento durante el transcurso de una comida/tetada
My baby had a big appetite	General Appetite	Apetito	Mi bebe tenía un gran apetito

[1] Response option: Never, Rarely, Sometimes, Often, Always. [2] Opción de respuestas: Nunca, Rara vez, Algunas veces, A menudo, Siempre.

Table S3. Full set of CEBQ-T and CEBQ-T-Mex items.

CEBQ-T [1]	Subescales		CEBQ-T-Mex [2]
**Food Aproach subscales; Sub-escalas pro-ingestión**			
My child is always asking for food<br>If allowed to, my child would eat too much<br>Given the choice, my child would eat most of the time<br>Even when my child has just eaten well, he/she is happy to eat again if offered	Food Responsiveness	Respuesta a los Alimentos	Mi hijo(a) siempre está pidiendo más comida<br>Si se le permitiera, mi hijo(a) comería demasiado<br>Si mi hijo pudiera escoger, se la pasaría comiendo la mayor parte del tiempo<br>Aun cuando mi hijo(a) haya comido bien, el/ella estaría contento(a) de volver a comer si se le ofreciera alimento

My child eats more when irritable<br>My child eats more when grumpy<br>My child eats more when upset	Emotional over-eating	Sobre-Alimentación Emocional	Mi hijo(a) come más cuando está enfadado(a)<br>Mi hijo(a) come más cuando esta malhumorado(a)<br>Mi hijo(a) come más cuando está molesto(a)
My child loves food<br>My child is interested in food<br>My child looks forward to mealtimes<br>My child enjoys eating	Enjoyment of Food	Disfrute de los Alimentos	A mi hijo(a) le encanta la comida<br>Mi hijo(a) está interesado en la comida<br>Mi hijo(a) espera con ganas la hora de las comidas<br>Mi hijo(a) disfruta comer
**Food Avoidance subescales; Sub-escalas anti-ingestión**			
My child has a big appetite*<br>My child cannot eat a meal if he/she has had a snack just before<br>My child leaves food on his/her plate or in the jar at the end of a meal<br>My child gets full before his/her meal is finished<br>My child gets full up easily	Satiety Responsiveness	Respuesta a la Saciedad	Mi hijo(a) tiene un gran apetito*<br>Mi hijo(a) no come a la hora de la comida si recibió un bocadillo justo antes<br>Mi hijo(a) deja alimentos en su plato o en su taza al final de una comida<br>Mi hijo(a) se siente lleno(a) antes de que termine su comida<br>Mi hijo(a) se siente lleno(a)/satisfecho(a), muy fácilmente
My child refuses new foods at first<br>My child enjoys a wide variety of foods*<br>My child enjoys tasting new foods*<br>My child is difficult to please with meals<br>My child decides that he/she does not like a food, even without tasting it<br>My child is interested in tasting food he/she has not tasted before*	Food Fussiness	Actitud remilgosa	Al principio mi hijo(a) rechaza nuevos alimentos<br>Mi hijo(a) disfruta de una gran variedad de alimentos*<br>A mi hijo(a) le gusta probar alimentos nuevos*<br>Mi hijo(a) es difícil de complacer con alimentos<br>Mi hijo(a) decide que no le gusta un alimento, antes de probarlo<br>A mi hijo(a) le interesa probar alimentos nuevos que no ha probado antes*
My child finishes his/her meal quickly*<br>My child eats slowly<br>My child takes more than 30 minutes to finish a meal<br>My child eats more and more slowly during the course of a meal	Slowness in Eating	Lentitud para comer	Mi hijo(a) termina sus alimentos rápidamente*<br>Mi hijo(a) es lento para comer<br>A mi hijo(a) le toma más de 30 minutos terminar sus alimentos<br>Mi hijo(a) come cada vez más lento durante el transcurso de una comida

[1] Response option: Strongly disagree, Disagree, Neither agree nor disagree, Agree, Strongly agree. [2] Opción de respuestas: Muy en desacuerdo, En desacuerdo, Ni en acuerdo ni en desacuerdo, En acuerdo, Muy en acuerdo.

Supplementary Information S2

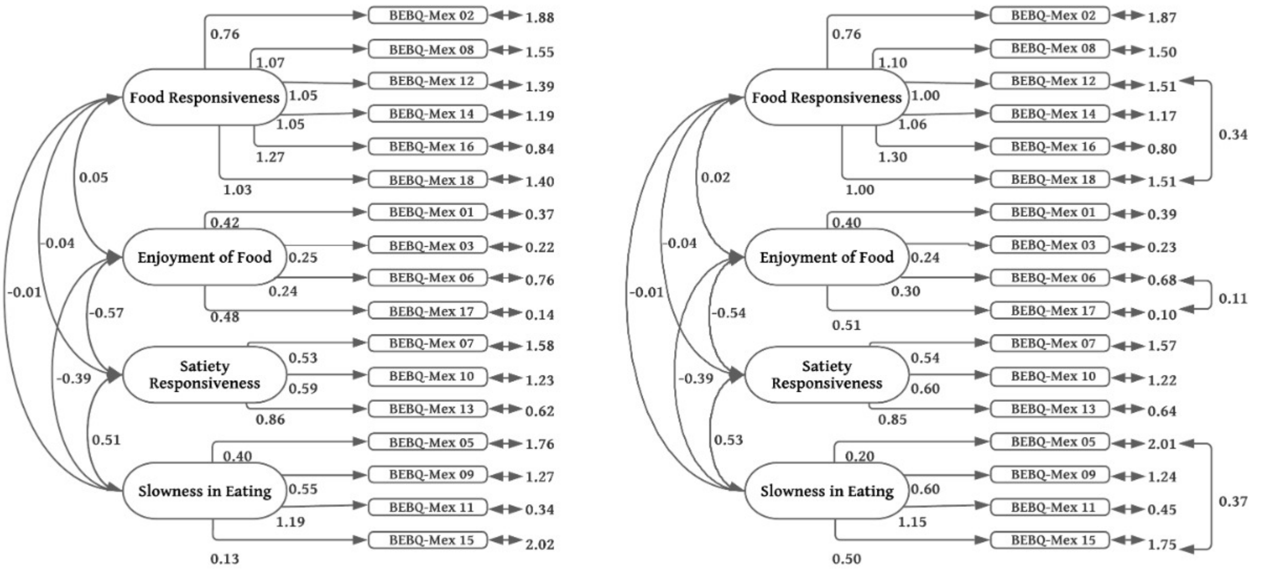

Figure S1. Path diagram of the four-factor models of the BEBQ-Mex with standardized estimates (factor-factor loadings, item squares multiple correlations and error-covariances) fitted in a sample of 330 mother-infant dyads (Model 1: left panel, Model 2: right panel).

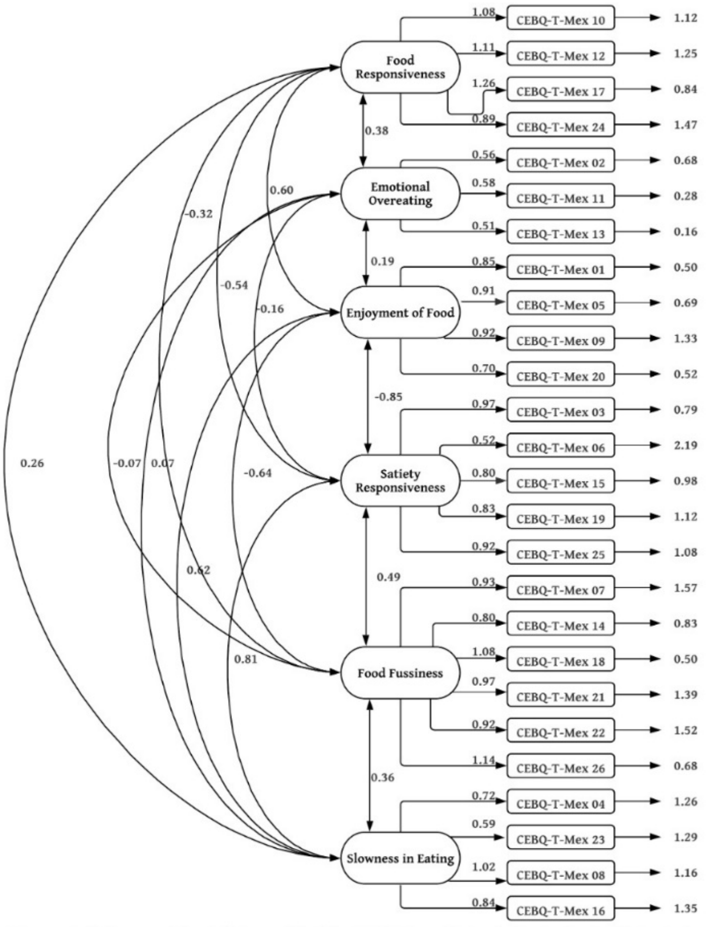

Figure S2. Path diagram of the six-factor model of the CEBQ-T-Mex with standardized estimates (factor-factor loadings, item squares multiple correlations and error-covariances) fitted in a sample of 330 mother-toddler dyads.

Results of the correlations between BEBQ-Mex subscales and BMI-z in infants revealed after adjusting for sex, age and feeding type, that those infants with greater response to food, had lower BMI-z, in direct opposition to results found in different studies [13,33,34] possibly pointing to the misinterpretation of the child's external cues [30]. This signals the need to address these issues and better educate mothers in a responsive feeding narrative [32]. Slowness in Eating, whose difficulties in showing good values of internal reliability, was negatively correlated to BMI-z after adjustment by age, sex and feeding type. These results have been replicated in several studies [13,23,33,34]. Infants with a more avid appetite tended to have a higher BMI-z. This last result was the only one observed in the Mexican study of 40 infants under the age of 6 months [17] (Table S4).

Toddlers with a greater enjoyment of food, who are unable to recognize their internal satiety cues and are not fussy eaters, tend to show increased weight. [36] have shown direct correlations with Food Responsiveness and inverse correlations with Satiety Responsiveness and Slowness in Eating and BMI-z scores in a prospective study of 210 infants followed prospectively from birth until 24 months of age in Singapore [36], similar to those found in our study. These results therefore suggest better interpretations of the toddlers' satiety cues by the mothers.

Table S4. Linear regressions between Infant and Toddler´s appetitive traits and BMI-z adjusting by sex, age and feeding type.

**Subscales**	**Infant BMI-z**		**Toddler BMI-z**	
	**β<br>95% CI**	**Adjusted-β [1]<br>95% CI**	**β<br>95% CI**	**Adjusted-β [1]<br>95% CI**
Food Responsiveness	−0.03 (−0.07, 0.12)	−0.002 (−0.10, 0.09)	0.02 (−0.10, 0.14)	0.04 (−0.08, 0.16)
Emotional Overeating	NA		0.05 (−0.02, 0.12)	0.05 (−0.02, 0.12)
Enjoyment of Food	0.03 (−0.01, 0.07)	0.02 (−0.01, 0.06)	0.091 (−0.003, 0.19)	0.13 (0.03, 0.22) **
Satiety Responsiveness	0.01 (−0.06, 0.09)	−0.01 (−0.09, 0.06)	−0.13 (−0.22, −0.04) **	−0.15 (−0.24, −0.05) **
Food Fussiness	NA		−0.03 (0.13, 0.08)	−0.07 (−0.17, 0.04)
Slowness in Eating	−0.05 (−0.12, 0.02)	−0.07 (−0.13, −0.001) *	−0.02 (−0.07, 0.04)	−0.03 (−0.08, 0.03)
General Appetite	0.10 (0.02, 0.18) *	0.09 (0.01, 0.17) *	NA	

[1] Ajusted by sex, age, feeding type. CI = Confidence Interval. * Correlation is significant at the 0.05 level (2-tailed). ** Correlation is significant at the 0.01 level (2-tailed).
